# Supplementary material for: Zebrafish Paralogs brd2a and brd2b Are Needed for Proper Circulatory, Excretory and Central Nervous System Formation and Act as Genetic Antagonists during Development
Source: J Dev Biol. 2021 Oct 31;9(4):46. doi: 10.3390/jdb9040046 (PMC8629005; doi:10.3390/jdb9040046)

SUPPLEMENTARY FIGURES

S1. Anti-Brd2b peptide antibody detects major protein products in zebrafish embryos and oocytes_ Western blot repeat of total ovary (ov) and staged embryos (4h, 24h, 48h) showing product between 50 and 75 kD in 24 and 48 hpf embryos, and faintly also in 4 hpf embryos.

A product over 150 kD is consistently detected in ovaries (ov) and 48 hpf embryos (shown here and in Figure 2) and sometimes faintly in 4 hpf and 24 hpf embryos (shown in Figure 2); a product between 50 and 75 kD is also consistently detected in 48 hpf embryos (shown here and in Figure 2) and sometimes also in 4 hpf and 24 hpf embryos (shown here).

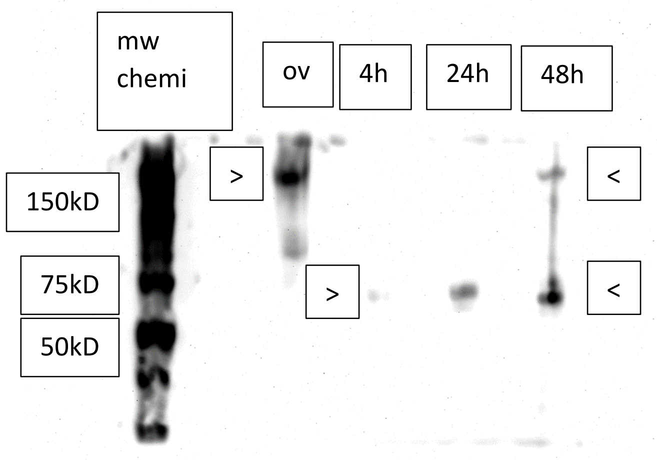

S2. Brd2b knockdown results in reduced hindbrain, ill-defined MHB region, and trunk abnormalities similar to Brd2a morphants, but presents unique circulatory and pronephric defects_HsBrd2RNA control

HsBrd2 RNA-injected embryo control:

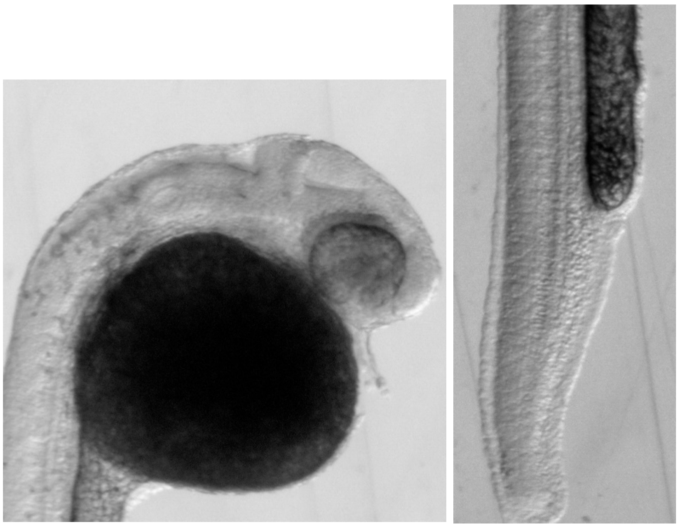

Midbrain-hindbrain boundary (MHB) region, pronephric duct cloaca, and peripheral blood island (PBI) are all normal in HsBrd2RNA-injected control embryos.

S3. Brd2b knockdown increases cell death in the CNS of prim 5 morphant embryos but reduces cell death in the cloaca of the pronephros_HsBrd2RNA control

TUNEL analysis on HsBrd2RNA-injected control embryos (head and trunk)

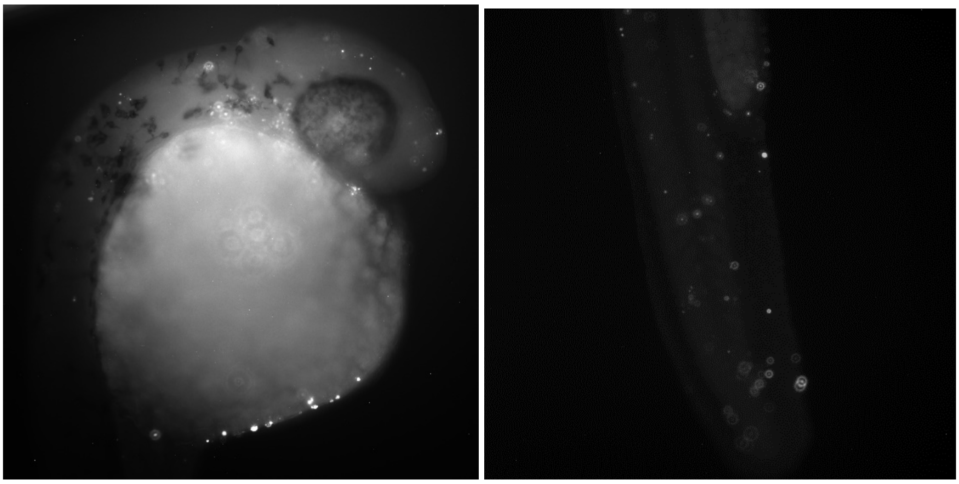

Supplement: Supplementary file 1 [file jdb-09-00046-s001.zip › jdb-1398518-supplementary.pdf]
